# Cell-Free Protein Synthesis From Fast-Growing *Vibrio natriegens*

**DOI:** 10.3389/fmicb.2018.01146

**Published:** 2018-06-01

**Authors:** Jurek Failmezger, Steffen Scholz, Bastian Blombach, Martin Siemann-Herzberg

**Affiliations:** Institute of Biochemical Engineering, University of Stuttgart, Stuttgart, Germany

**Keywords:** *in vitro* translation, *Vibrio natriegens*, ribosomes, rRNA, cell-free extract

## Abstract

*Vibrio natriegens* constitutes one of the fastest-growing nonpathogenic bacteria and a potential novel workhorse for many biotechnological applications. Here, we report the development of a *Vibrio*-based cell-free protein synthesis system (CFPS). Specifically, up to 0.4 g L^-1^ eGFP could be successfully synthesized in small-scale batch reactions using cell-free extract obtained from fast-growing *V. natriegens* cultures. Versatile CFPS system characterization attained by combining the analyses of key metabolites for translation and ribosomes revealed limitations regarding rRNA stability and critical substrate consumption (e.g., amino acids). Alternatively, rRNA showed increased stability by inducing Mg^2+^homeostasis in the reaction. Although the enormous translation capacity of the CFPS system based on the available ribosome concentration could not yet be fully exploited, its potential was successfully demonstrated by activating an endogenous transcription unit with *V. natriegens*RNA polymerase (RNAP) for protein expression. This allowed the use of *in vitro* screening for promoter strength, a critical factor for efficient gene expression *in vitro* and *in vivo*. Three different promoters were tested and output signals corresponded well with the expected affinity for *V. natriegens* RNAP. This established CFPS toolbox may provide a foundation to establish *V. natriegens* as a valuable platform in biotechnology as well as synthetic biology.

## Introduction

Recent studies highlight the potential of fast-growing bacteria to speed up lab routines and biotechnological processes (Cordova et al., 2015; Weinstock et al., 2016; Hoffart et al., 2017). Among such bacteria, the Gram-negative γ-proteobacterium *Vibrio natriegens* represents a promising candidate that possesses remarkably high growth and substrate consumption rates (Weinstock et al., 2016; Hoffart et al., 2017; Long et al., 2017) as prerequisite to develop fermentation processes operating with very high productivity (Hoffart et al., 2017) Furthermore, genetic engineering tools for *V. natriegens* have been developed and allow the directed manipulation of its cellular metabolism (Weinstock et al., 2016; Hoffart et al., 2017) and heterologous protein production (Weinstock et al., 2016). In particular, inducible promoter systems (e.g., *lacUV5, trc*, and *araBAD*) have been validated for the latter to drive expression of *gfp* in *V. natriegens* (Weinstock et al., 2016).

*V. natriegens* grows rapidly in minimal medium supplemented with various carbon sources under both aerobic and anaerobic conditions (Hoffart et al., 2017). However, exceptionally fast growth was observed in complex medium (Eagon, 1962; Weinstock et al., 2016; Hoffart et al., 2017) reaching differential growth rates of up to 4.43 h^-1^, which corresponds to a doubling time (t_d_) of 9.4 min (Hoffart et al., 2017). Aiyar et al. (2002) showed that with increasing growth rate the increase in ribosome number in *V. natriegens* accelerates yielding an estimated number of 115,000 ribosomes per cell at a t_d_ of approximately 15 min. This number is significantly higher compared to 70,000 ribosomes per cell in *Escherichia coli* at a t_d_ of 25 min and also outcompetes the extrapolated value of 90,000 ribosomes per cell at a t_d_ of 15 min (Aiyar et al., 2002). These characteristics support that *V. natriegens* may have potential as a platform for cell free protein synthesis (CFPS).

Cell-free systems, originated from crude cell extracts predominantly derived from *E. coli*, have been successfully used to discover the mechanisms of life, such as deciphering the genetic code or protein synthesis (for a recent review see Moore et al., 2017b). This technology has also been established in many fields of applied biotechnology, thereby enabling new applications in genetic prototyping and biomanufacturing. CFPS has been applied as well in the upcoming disciplines of synthetic biology, such as for the rapid prototyping of biological circuits and metabolic pathways with the long-term goal of using CFPS for natural product discovery (Takahashi et al., 2015; Jiang et al., 2018). Moreover, the design of paper-based cell-free diagnostics (Pardee et al., 2014, 2016) broadens its potential application in pharmaceutical diagnostics. In particular, because of the absence of barriers such as the cell wall or membranes, the cell-free reaction environment is open and therefore accessible and controllable, thus allowing direct and easy manipulation, monitoring, sampling, and optimization (Jewett et al., 2008). Notably, these features idealistically encompass basic experimental requirements; e.g., for detailed reaction analysis in systems biology (Niess et al., 2017).

Recently some *E. coli*-alternative CFPS systems were successfully introduced. *Streptomyces*-based systems were reported to address antibiotic production-related issues (Li et al., 2017; Moore et al., 2017a), and certain synthetic biology aspects (promoter-studies) were addressed using *Bacillus subtilis* as a core system (Kelwick et al., 2016). Moreover, several systems based on crude extract from eukaryotic cells, namely from *Saccharomyces cerevisiae* (Hodgman and Jewett, 2013; Gan and Jewett, 2014), rabbit reticulocytes (mammalian) (Anastasina et al., 2014), and Chinese hamster ovary (CHO, mammalian) cells (Brödel et al., 2014) were established. These systems offer the advantage of various degrees of posttranslational modifications but are restricted to low productivity, most likely owing to the rather low level of translationally active compounds such as ribosomes. This suggests thathighly productive protein synthesis may instead be compiled using ultra-fast-growing (non-pathological) microbes, targeting *Vibrio* as a most promising candidate considering its unparalleled growth rate and high ribosome number. In addition, a robust cell-free *Vibrio*-based system may also serve as a promising platform to complement existing strategies for pathway discovery. In this context, we present the development and characterization of a CFPS system using crude extract derived from *V. natriegens.*

## Results and Discussion

### *Vibrio natriegens* Supports CFPS

**FIGURE 1:**
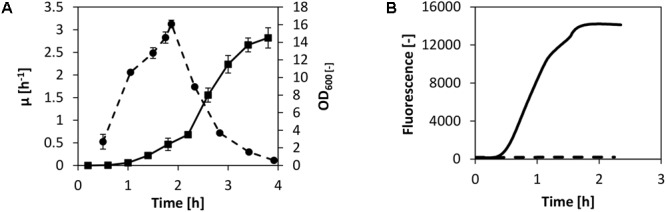
CFPS from *V. Natriegens*. **(A)** Shake flask cultivation of *V. natriegens* in BHIN media at 37°C. Depicted are the biomass (solid line) and the differential specific growth rate (dashed line). For lysate preparation, cells were harvested at maximum growth. Error bars denote standard deviations of three independent measurements. **(B)** Cell-free protein synthesis of eGFP using *V. natriegens* cell-free extract (solid line). The negative control without plasmid is also shown (dashed line).

Our goal was to set up a CFPS system using fast-growing *V. natriegens* biomass by adopting the well-established protocols available for *E. coli in vitro* translation. These protocols usually consist of biomass cultivation via a bioreactor or shake flask, harvest of the biomass during fast growth, cell lysis via high-pressure homogenization, high-speed centrifugation, run-off reaction, dialysis, and a final centrifugation step (Pratt, 1984; Nevin and Pratt, 1991; Liu et al., 2005). Even though we have recently demonstrated active cell-free extract from non-growing cells (Failmezger et al., 2017b), we decided to utilize the traditional cell-free extract preparation method as described above. An exception was the growth medium: instead of using 2× YTPG, which is the most applied media for cell-free translation systems using *E. coli, V. natriegens* was cultivated in BHN (brain–heart infusion with NaCl). In this manner, high specific growth rates up to 3 h^-1^ were obtained in shake flask cultivations performed at 37°C (**Figure 1A**).

The biomass was harvested at maximum growth after only 2 h of cultivation and cell-free extract was prepared as described earlier in the text. To test the potential of *in vitro* translation for the cell-free extract from *V. natriegens*, we commenced by performing cell-free protein synthesis of eGFP. We used the identical reaction setup as described in our previous report for *E. coli* (Failmezger et al., 2016). In detail, transcription of eGFP was controlled by a T7 promoter and the reaction was spiked with T7 RNA polymerase (RNAP). Concentrations of substrates (amino acids and nucleotides), salts, and buffer components were also adopted. Exogenous energy regeneration by creatine phosphate and creatine kinase was used to supply the system with regenerated ATP and GTP. The reaction was carried out in a scale of 250 μL at 37°C, and eGFP synthesis was monitored online. Notably, combined transcription and translation in the *V. natriegens* system was confirmed by eGFP synthesis as demonstrated in **Figure 1B**. Expression of eGFP was detected after 30 min of incubation and was accompanied by a linear increase of fluorescence over the course of about 1 h. Active synthesis was followed by a decline of the synthesis rate and a termination of the reaction after a total reaction time of 2 h.

### Characterization of the *V. natriegens* Cell-Free Extract

Upon demonstration of active protein synthesis by a cell-free translation system based on *V. natriegens*, our aspiration was to examine and characterize the system. As ribosomes denote the key for translation, we aimed to quantify the rRNA concentration in the extract. Extraction and quantification of rRNA revealed a concentration of 113 ± 6 g L^-1^ rRNA (sum of 16S and 23S) in the cell-free extract from *V. natriegens*. This concentration is roughly four-times higher than that in our standard *E. coli* cell-free system and mirrors the observed fast growth of *V. natriegens*. Moreover, the measured rRNA concentration correlates nicely with the approximately four-fold higher specific growth rate between *V. natriegens* and *E. coli* at the point of cell harvest and is consistent with the general consensus that *V. natriegens* achieves fast growth through increased ribosome numbers (Aiyar et al., 2002). In addition, as we always resuspended 1 g biomass (wet weight) in 1 mL lysis buffer prior to homogenization, the direct comparison of certain characteristics, such as the ribosome concentration between both systems, is justified.

CFPS depends on highly active catalytic proteins provided by the cell-free extract. However, although we have previously shown that the cell-free extract preparation procedure preserves crucial components involved in translation in the case of *E. coli* (Failmezger et al., 2016), measurement of the rRNA integrity in the *V. natriegens* system before and after the run-off incubation at 37°C, which can be regarded as the most critical step of the process, revealed no alteration in the rRNA integrity or concentration (Supplementary Figure S1). Hence, we concluded that similar to *E. coli*, lysate processing conditions do not detrimentally affect the catalyst composition.

### Efficient Energy Regeneration by Exogenous but Not Endogenous Systems

**FIGURE 2:**
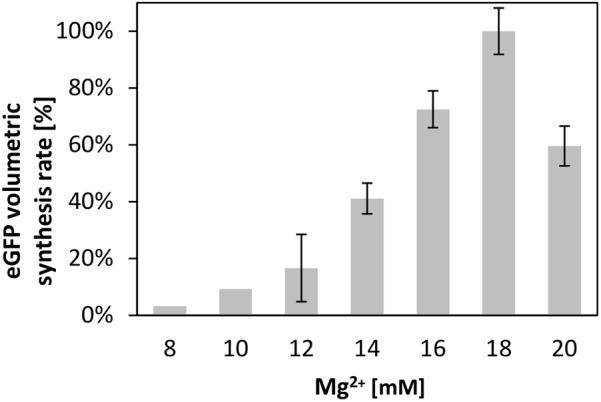
Cell-free protein synthesis energized by creatine phosphate and creatine kinase. Differential rate of eGFP synthesis over time at different magnesium concentrations. The maximum of 100% corresponds to 1.33 ± 0.1 mg_eGFP_× L^-1^ × min^-1^. Error bars represent standard deviations of triplicate measurements.

Supplying the transcription and translation reactions with energy is crucial for efficient protein synthesis (Kim and Swartz, 1999; Kim and Kim, 2009). Therefore, we compared several of the most common energy regeneration systems, namely creatine phosphate in combination with creatine kinase, phosphoenolpyruvate (PEP), and pyruvate, for their ability to energize the translation reaction in the *V. natriegens* CFPS system. For each system, we optimized the magnesium ion concentration, a parameter well known to impact the translation performance (Kim and Choi, 2000; Failmezger et al., 2016). Similar to cell-free translation systems from other source organisms; e.g., from *B. subtilis* (Kelwick et al., 2016), we found that the magnesium concentration heavily influenced the synthesis rate in the CFPS from *V. natriegens*. This is demonstrated in **Figure 2**, which illustrates the relative synthesis rate at different concentrations of magnesium at a constant concentration of 60 mM creatine phosphate. A clear optimum at 18 mM magnesium was achieved. Notably, the increase of the maximum rates, as well as the eGFP titer, both correlated linearly with the Mg-glutamate concentration until the optimum was reached (*R*^2^ = 0.95 or *R*^2^ = 0.93 for a linear regression).

Next, we investigated the potential of endogenous energy regeneration by supplying the reaction with PEP or pyruvate. We observed active translation using PEP; however, the eGFP synthesis rates were markedly decreased compared to those of creatine phosphate (Supplementary Figure S2A). A similar scenario was obtained using pyruvate to regenerate ATP, with similar final titers and maximum synthesis rates also being obtained (Supplementary Figure S2B). Thus, it can be concluded that whereas energy regeneration by endogenous enzymes in the *V. natriegens* extract is possible; this strategy is less productive than exogenous energy regeneration by creatine kinase. This may be due to the high activity of metabolic pathways unfavorably channeling glycolytic substrates away from ATP synthesis. Our finding is similar to that observed in other cell-free systems, such as upon blocking the conversion of pyruvate to PEP by phosphoenolpyruvate synthetase using oxalate increased productivity in an *E. coli* CFPS system (Kuem et al., 2006). Whether a similar increase in productivity can be achieved in the *V. natriegens* system, for example by blocking certain metabolic pathways, remains to be investigated.

### Prevalent Amino Acid Degradation in the *V. natriegens* CFPS system

**FIGURE 3:**
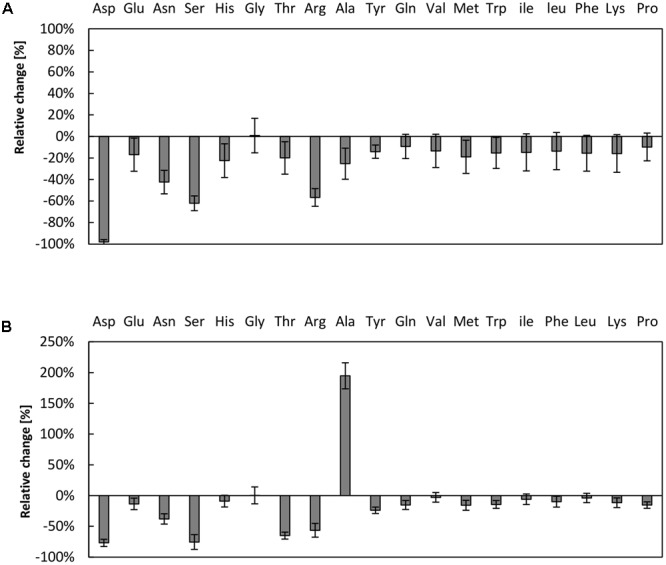
Amino acid degradation in the *V. natriegens* CFPS system. Relative concentrations of amino acids after 3 h of CFPS fueled by **(A)** creatine phosphate or **(B)** PEP. Initial concentrations were set to 1 mM of each amino acid. Note that cysteine could not be reliably measured using our analysis method. Error bars represent standard deviations of triplicate measurements.

The supply of the translation reaction with amino acids has previously been shown to be critical in cell-free systems (Kim and Choi, 2000; Jewett and Swartz, 2004b; Calhoun and Swartz, 2006; Kim et al., 2006). A bottleneck in the amino acid supply can partially be overcome by raising the amino acids concentration up to 2 mM (Kim and Swartz, 2001) or by designing strains lacking several amino acid degradation pathways (Calhoun and Swartz, 2006). As it can be assumed that amino acid degradation is prevalent in our reaction, we investigated the amino acid stability in the CFPS system from *V. natriegens*. Therefore, samples were taken after 3 h of reaction time and the amino acids content was determined via high performance liquid chromatography (HPLC) analysis and compared to initial conditions. In the reaction system empowered by creatine phosphate, the three amino acids aspartic acid, serine, and arginine were almost entirely consumed (**Figure 3A**). Notably, aspartic acid was entirely depleted in the reaction, whereas more than half of the amounts of serine and arginine were degraded. A similar scenario was obtained for the system fueled by PEP, wherein it was also demonstrated that aspartic acid, serine, and arginine could be regarded as most critical (**Figure 3B**). Moreover, the amount of alanine doubled in the reaction fueled by PEP. It may be hypothesized that several enzymatic activities are responsible for the observed turnover of amino acids. For example, it was previously demonstrated that arginine depletion was due to arginine decarboxylase activity in a CFPS system from *E. coli* (Calhoun and Swartz, 2006). In addition, it was claimed that the decrease of serine is associated with pyruvate formation by serine deaminase (Calhoun and Swartz, 2006). It is therefore likely that similar enzymatic activities are present in the cell-free extract from *V. natriegens* as in *E. coli*, indicating a similar core metabolism as has been recently demonstrated (Long et al., 2017).

### Transcription Template Source Impacts Translational Performance

**FIGURE 4:**
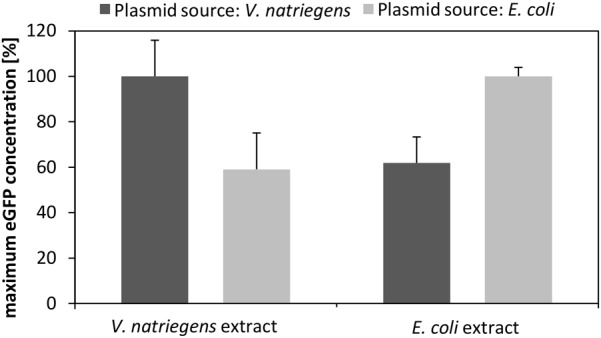
CFPS performed with *V. natriegens* cell-free extract and identical plasmids prepared from *V. natriegens* or *E. coli*. The same experiment was also performed with *E. coli* extract and plasmids prepared either from *V. natriegens* or from *E. coli*. The maximum eGFP concentration obtained after 3 h of an in vitro translation reaction is shown. Plasmid concentrations were kept constant for all experiments.

For transcription of eGFP we used our standard expression plasmid, which we prepared from *E. coli* DH5α. However, to test whether the plasmid source affected the transcriptional and translational performance, we purified the plasmid from *V. natriegens* and compared the performance of CFPS reactions with that using the plasmid prepped from *E. coli*. As depicted in **Figure 4**, the source of the plasmid markedly affected the translation performance. Specifically, the *V. natriegens* CFPS system performed 40% better in the case where the plasmid was also prepared from *V. natriegens*. Notably, the control experiment using our established *E. coli* CFPS system with the plasmids from each source confirmed our findings. It can be hypothesized that differences in the restriction-modification system between both species impact the plasmid stability and subsequently the transcription and translation performance. This is emphasized by the fact that the transformation efficiency of *V. natriegens* is reduced if the plasmid is isolated from a different source organism (e.g., *E. coli*) (Weinstock et al., 2016). Therefore, it could be concluded that it is essential that the expression plasmid have the same cellular background as the CFPS systems to which it is applied.

### The *V. natriegens* Core RNAP Can Be Recruited for CFPS *in Vitro*

**FIGURE 5:**
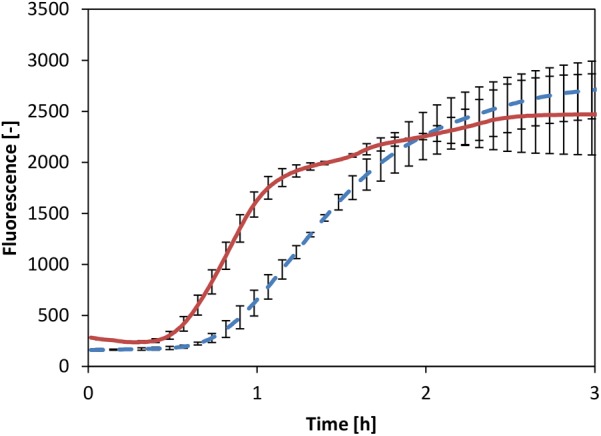
CFPS with T7 RNAP (dotted line) and core RNAP (solid line). Error bars represent standard deviations of triplicate measurements.

A powerful alternative to the common transcription strategy by exogenous T7 RNAP is the recruitment of the core RNAP, which comprises part of the cell-free extract. It has been demonstrated that the core RNAP together with the housekeeping factor σ^70^ acts as a powerful transcription unit in a CFPS system based on *E. coli* (Shin and Noireaux, 2010). Therefore, we chose to test whether a similar transcription unit could be activated in our *V. natriegens* system. Thus, the reaction was supplemented with a readily available plasmid in which eGFP expression was controlled by the σ^70-^dependent *Pr* promoter originating from *E. coli*. Considering the fundamental similarities between the two systems described above, we reasoned that this promoter sequence could also function in the *V. natriegens* system. Comparison of CFPS by the core RNAP to the system in which mRNA was generated by T7 RNAP showed identical titers of eGFP (**Figure 5**). Notably, reaction kinetics appeared to be faster in the system where mRNA was generated by the core RNAP. Conversely, it was previously demonstrated that the T7 RNAP is quicker than the core RNAP, at least in reported CFPS systems based on *E. coli* extract, resulting in an uncoupling of transcription and translation (Iskakova et al., 2006). However, we considered that the use of core RNAP might lead to extended synchronization of transcription and translation events, thus enabling more efficient translation in earlier reaction times, as was observed. Overall, this experiment clearly demonstrated that the *V. natriegens* core RNAP could be recruited to enable protein expression *in vitro*.

### rRNA Stability in the CFPS Reaction Requires Magnesium Homeostasis

**FIGURE 6:**
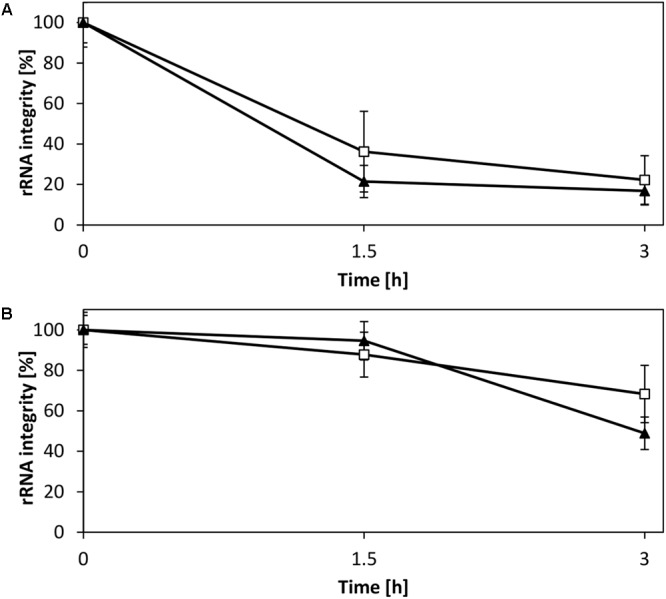
Stability of rRNA in CFPS reactions. Shown is the integrity of the 16S rRNA (□) and 23S rRNA (▼). **(A)** CFPS fueled with creatine phosphate, which results in the accumulation of phosphate and magnesium sequestration. **(B)** CFPS fueled with pyruvate allows for magnesium homeostasis and demonstrates elevated rRNA stability. Error bars represent standard deviations of triplicate measurements.

rRNA is a potential target for degradation in the CFPS reaction owing to sequestration of magnesium ions, as previously demonstrated for a CFPS system from *E. coli* (Failmezger et al., 2016). This motivated us to evaluate the rRNA stability in the *V. natriegens* system. Analysis of the rRNA in a CFPS reaction fueled with creatine phosphate demonstrated a decay of intact 16S and 23S rRNA (**Figure 6A**), consistent with the accompanying decrease of magnesium as shown in our previous study (Failmezger et al., 2016). In contrast, providing magnesium homeostasis by using pyruvate resulted in elevated stability of both rRNA species (**Figure 6B**). Based on these observations, it was evident that the CFPS system from *V. natriegens* is prone to similar rRNA degradation mechanisms as reported for *E. coli* (Failmezger et al., 2016). However, although the 16S rRNA represented the main target for degradation in the *E. coli* CFPS system, the experimental results presented here suggested an equal decay of both the 16S and the 23S rRNA. Moreover, the electropherograms revealed no cleavage products (Supplementary Figure S3); therefore, it could be hypothesized that the rRNA degradation can be characterized by an exonuclease activity. Thus, our efforts clearly demonstrated that magnesium also constitutes a key parameter for efficient translation and preservation of ribosome integrity in the *V. natriegens* CFPS system.

### Optimized Reaction and Scale Down

**FIGURE 7:**
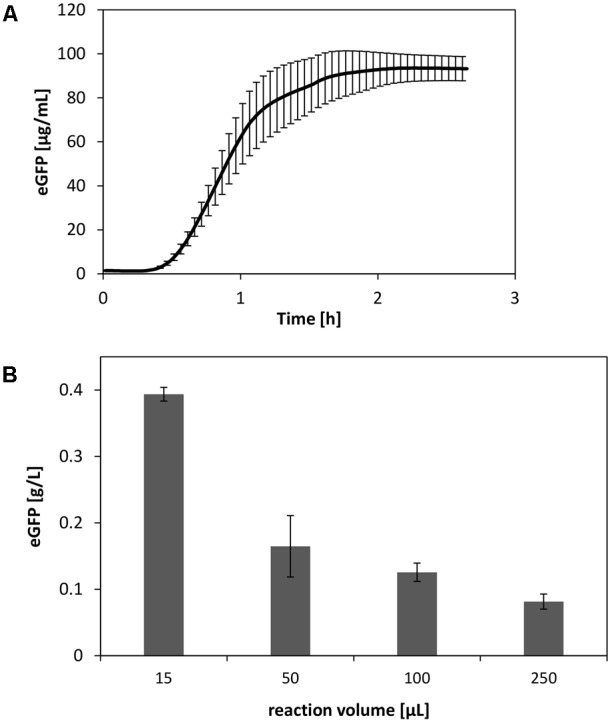
**(A)** Optimized CFPS reaction of eGFP at a scale of 250 μL. **(B)** Scale down of the CFPS reaction performed in reaction tubes. Error bars represent standard deviations of triplicate measurements.

Using the insights obtained regarding the *V. natriegens* CFPS system, we performed cell-free synthesis of eGFP under optimized reaction conditions. This included, e.g., elevated concentrations of amino acids, optimized magnesium levels, and plasmid prepped from *V. natriegens*. In this manner, we were able to synthesize up to 90 μg/mL eGFP at a reaction scale of 250 μL (**Figure 7A**). However, it is evident that the reaction scale limits the productivity of CFPS systems (Voloshin and Swartz, 2005). Therefore, by scaling down the reaction, final product titers could be further increased up to four-fold (**Figure 7B**).

From this experiment, it was clear that the performance levels achieved thus far did not meet the expectations with respect to the measured high ribosome concentration in the extract. Although bulk protein synthesis rates for published *E. coli* CFPS systems are in the range of 6–10 mg L^-1^ min^-^1 (Niess et al., 2017), a three- to fourfold higher ribosome concentration, as we detected in the *V. natriegens* cell-free extract, should allow for rates in the range of up to 40 mg L^-1^ min^-1^, resulting in theoretical protein levels of several grams per liter after standard reaction times. Consequently, owing to the observed still low volumetric synthesis rates, it might be inferred that only a partial fraction of the ribosomes could be activated under the current reaction conditions. Therefore, further strategies to improve the performance and to unleash the theoretical power of the system needed to be developed. In this regard, following the already established guiding principle of advancing CFPS by cytoplasmic mimicry (Jewett and Swartz, 2004a; Jewett et al., 2008) is expected to enable highly improved performance levels of CFPS by *V. natriegens*.

Notably, recent reports propose a similar scenario for *in vitro* translation systems from *E. coli*, wherein it was demonstrated that ribosomes were only partially actively participating in translation (Failmezger et al., 2017b; Kempf et al., 2017). Hence, it might be suggested that limited ribosome activity represents a general bottleneck in CFPS.

### CFPS From *V. natriegens* Enables Screening for Promoter Strength

**FIGURE 8:**
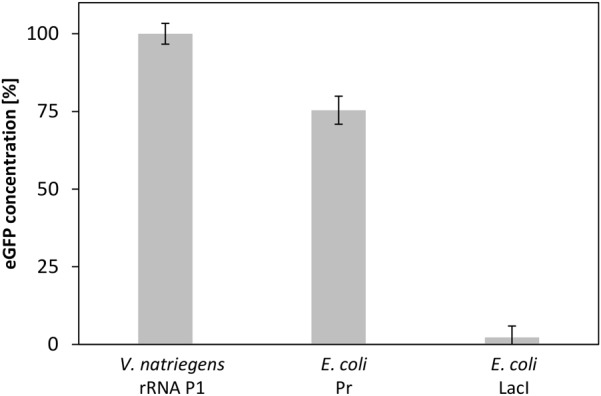
CFPS of eGFP with *V. natriegens* extract using different promoter elements. The strong promoters *rRNA P1* from *V. natriegens* and *Pr* from *E. coli*, and *pLacI* from *E. coli* as a weak promoter were used. Error bars represent standard deviations of triplicate measurements.

A valuable application of CFPS systems is the rapid screening for protein expression using different regulation entities (Sawasaki et al., 2002). As *V. natriegens* represents a potential host organism for the biotechnology industry (Hoffart et al., 2017), powerful expression systems need to be developed. A step toward this goal is the identification of strong promoters to allow efficient gene expression. To demonstrate the potential of our developed CFPS system to enable the identification of possible promoters of *V. natriegens*, we established a small promoter library consisting of a set of plasmids with three different promoter elements. Although little is known regarding promoters from *V. natriegens*, a promoter sequence for ribosomal protein P1, which is considered to be strong, has already been published (Aiyar et al., 2002). Lacking any information of comparable weak promoters, we relied upon the weak promoter P_lacI_ from *E. coli*. In addition, the strong σ^70^-dependent *Pr* promoter from *E. coli* was also included in the experiment. Screening this small library showed three distinct output signals (**Figure 8**). Whereas the highest eGFP expression was obtained for the strong *V. natriegens* promoter, a decreased synthesis level was detected for the σ^70^ promoter from *E. coli*, and almost no expression was obtained for the weak *E. coli* promoter. This experiment clearly demonstrated the ability to screen for promoter strength in a straightforward and rapid manner using our established CFPS system from V. *natriegens*.

## Conclusion

*V. natriegens* constitutes a remarkably fast-growing bacterium that shows potential to becoming the next platform for biotechnology (Hoffart et al., 2017; Long et al., 2017). A CFPS system based on *V. natriegens* may facilitate the effective utilization of this organism for diverse applications in the fields of industrial biotechnology and synthetic biology. Herein, we reported the development of a *V. natriegens* cell-free system able to synthesize the model protein eGFP. Thorough characterization revealed the enormous potential of this system to synthesize proteins based on the measured concentration of rRNA in the cell-free extract. However, further improvements of the reaction setup and the screening of factors currently limiting the overall performance of the system, e.g., ribosome activity, is needed to exploit the full potential of this cell-free translation system. Towards this end, the application of *Vibrio* specific components such as tRNA, a detailed component optimization beyond *E. coli* specific routines (e.g., mono-and bivalent ions, buffer-specific items) and a *Vibrio*-specific lysate preparation routine will be initiated.

Whereas these limitations will be addressed in future work, the applicability of our *V. natriegens* cell-free protein synthesis system was successfully demonstrated by screening for potential genetic regulatory elements. Looking forward, we believe that our study represents the first step toward the establishment of a high yielding CFPS system based on extract from *V. natriegens*.

## Materials and Methods

### Culture Condition and Biomass Generation

*Vibrio natriegens* was stored at -70°C as a glycerol stock (30% vol/vol). For cultivation, the stock was streaked on brain–heart infusion (BD Bactol; 37 g L^-1^) agar (18 g L^-1^) plates supplemented with 15 g L^-1^ NaCl (BHIN-medium), and incubated overnight. A single colony was used to inoculate the preculture I (5 mL BHIN-medium in a test tube. Preculture I was cultivated at 37°C on a rotary shaker at 120 rpm until the stationary phase was reached. Preculture II (100 mL BHIN in a 500-mL baffled Erlenmeyer flask) was inoculated with preculture I (1/100) and cultivated for 2.5 h overnight at 37°C on a rotary shaker at 120 rpm. The main culture (400 mL BHIN medium in a in a 2000-mL baffled Erlenmeyer flask) was inoculated to a cell density of 0.1. During the cultivation, samples were taken and cell density was determined at 600 nm using a photometer. The differential growth rate μ_diff_ was calculated using formula (1). When the exponential phase was reached the biomass was rapidly chilled by incubation in an ice bath. Cell were harvested by centrifugation (5000 × *g* for 20 min at 4°C). Biomass was flash frozen and stored at -70 °C until cell lysis

(1) $$\begin{array}{c} \mu_{\mathrm{diff}}\;=\;\frac{1}{t2\;-\;t1}\;\times \;\ln\;\left(\frac{OD2}{OD1}\right) \end{array}$$

### Cell-Lysate Preparation

Cell free lysate of *V. natriegens* was prepared according to the standard protocol that is applied in our laboratory to prepare *E. coli* cell free lysate. All steps aside from the run-off reaction were performed at 4°C. The frozen biomass was thawed in 1 mL cold S30 buffer per g biomass [14 mM magnesium acetate, 60 mM potassium acetate, 10 mM Tris, pH 8.2, 2 mM dithiothreitol (DTT)]. The cell suspension was lysed by passage through a high-pressure homogenizer (Emulsi-Flex-C5, Avestin, Canada) at 12,000 kPa. The lysate was clarified by two centrifugation steps at 30,000 × *g* for 30 min at 4°C. Subsequently, the cell lysate was incubated at 37°C for 80 min at 120 rpm on a shaker, for the run-off reaction. The cell-free extract was then dialyzed against a 100-fold larger volume of S30 dialysis buffer (14 mM magnesium acetate, 60 mM potassium acetate, 10 mM Tris, pH 8.2, 1 mM DTT) for 4 h at 4°C. After a centrifugation at 4000 × *g* for 20 min at 4°C, the clear cell lysate was aliquoted, flash frozen in liquid nitrogen, and stored at -70°C.

### CFPS

The CFPS reaction was performed at a volume of 250 μL in a 96-well plate. The standard reaction mixture consisted of the following components: 24% (v/v) S30 cell lysate, 36 μg mL^-1^ plasmid-DNA, 1.2 mM of each amino acid, 18 mM Mg-glutamate, 20 mM ammonium-glutamate, 34 μg mL^-1^ folinic acid, 2% (w/v) PEG (8000) 80 mM HEPES-KOH (pH 8.0), 60 mM creatine phosphate, 1.2 mM ATP, 1 mM each GTP, CTP, and UTP, 2 mM DTT, 240 μg/mL creatine kinase, and 1 U/ μL T7 RNAP (Roche Diagnostics, Mannheim, Germany). The expression of eGFP was monitored online by fluorescence (excitation filter 485 nm, emission 520 nm) in a Synergy 2 plate reader (Biotek Instruments, Winooski, VT, United States) at 37°C. Prior to each reading cycle the plates were shaken. When PEP (60 mM) or pyruvate (60 mM) was used as an energy source, 0.32 mM NAD and 0.32 mM CoA were added to the reaction. The Mg-glutamate concentration was optimized for each reaction condition by performing an optimization routine consisting of a stepwise increase of Mg^2+^ in 2 mM increments The reaction was downscaled in polymerase chain reaction (PCR)tubes.

### rRNA Extraction and Analysis

The extraction of the total RNA and the analysis of the rRNA from the cell lysate using capillary gel electrophoresis with laser-induced fluorescence detection was performed as described previously (Failmezger et al., 2016, 2017a).

### Analytics

To quantify amino acids during the cell free reaction, samples were taken after 0, 90, and 180 min. The samples were diluted 1/20 and heated to 90°C for 5 min to stop all biochemical reactions. Precipitated proteins were removed by centrifugation (5 min at 5000 × *g* and 4°C) to obtain a clear supernatant.

L-amino acids were analyzed by reversed-phase HPLC using an Agilent 1200 Series apparatus (Agilent Technologies, Santa Clara, CA, United States) equipped with an Agilent ZORBAX Eclipse Plus C18 column (250 × 4.6 mm, 5 μm) protected by a 286 Agilent ZORBAX Eclipse Plus C18 guard column (12.5 × 4.6 mm, 5 μm) and a fluorescence detector at 40°C. The mobile phase consisted of the two eluents: Buffer A (10 mM Na_2_HPO_4_, 10 mM Na_2_B_4_O_7_, 0.5 mM NaN_3_, pH 8.2) and Buffer B [B; 0.1 M KH_2_PO_4_, 0.1 M K_2_HPO_4_, 4 mM tetrabutylammonium sulfate, pH 7.2, 30% (v/v) methanol]. Before injection, the amino acids were automatically pre-column derivatized with ortho-phthaldialdehyde and fluorenylmethoxycarbonyl chloride. The following gradient was generated at a flow rate of 1.5 mL min^-1^: 0% B; 0.84 min, 50% B; 33.4 min, 0% B; 33.5 min, 0% B 39.3 min; 0% B; 39.4 min, 0% B; 40 min. Detection of the derivatized L-amino acids occurred via a fluorescence detector, with different retention times corresponding to the single derivatized L-amino acids. The L-amino acids were quantified using an 8-point calibration curve for each amino acid as an external reference standard and by using L-ornithine as an internal standard.

### Plasmids

The Plasmid pJOE4056.2 (kindly provided by J. Altenbuchner, IIG, University of Stuttgart), containing an eGFP gene between a T7-Promotor and a T7-terminator, was used for the expression of eGFP from T7-RNAP. The plasmids pJOE 4052.2 (kindly provided by J. Altenbuchner, IIG, University of Stuttgart) and pBEST-OR2-OR1-Pr-UTR1-deGFP-T500 which was a kind gift from Vincent Noireaux (Addgene plasmid # 40019) (Shin and Noireaux, 2010) were used for the expression of eGFP from the *V.natriegens* core RNAP and σ^70^ factor, respectively. All three plasmids contain an *ampR* marker for selection.

Furthermore, the *Pr* promoter of pBEST-OR2-OR1-Pr-UTR1-deGFP-T500 was replaced by the weak promoter *PLacI* or the strong promoter *rRNAP1* using the restriction enzymes RSphI and NheI. The promoter sequences were amplified from genomic DNA of *V. natriegens* and *E. coli* by PCR. Restriction sites for RSphI and NheI were added using the following primer pairs (5′–3′): CTG GGC ATG CTA GGG GTA AAG TTG GAT AAA TAA and TAT TGC TAG CCT TCG GGA GAG GCG GCC (rRNA P1); and CTG GGC ATG CAT CGA ATG GCG CAA AAC C and TAT TGC TAG CTC ATT CAC CAC CCT GAA TTG (PLacI).

### DNA Template Preparation

The plasmids were transformed into *V. natriegens* cells by electroporation and plated on BHIN-ager. Ampicillin (100 μg mL^-1^) was used for selection during the cultivation. BHIN (5 mL) was inoculated with a single colony and incubated at 37°C and 120 rpm until the stationary phase was reached. Then, 400 mL BHIN in a 2-L shake flask was inoculated 1/1000 and incubated at 37°C and 120 rpm overnight until the stationary phase was reached. The biomass was collected by centrifugation at 5000 × *g* at 4°C for 20 min. The DNA template for protein expression was purified using the NucleoBond^®^ Xtra Maxi Kit (Macherey-Nagel GmbH & Co., KG, Düren, Germany) according to manufacturer instruction. The DNA concentrations were measured using a NanoDrop instrument (Wilmington, DE, United States). DNA solution was diluted to 900 μg mL^-1^, aliquoted, and stored at -20 °C.

## Author Contributions

JF designed the study, analyzed the data, wrote and drafted the manuscript. SS performed the experiments and analyzed the data. SS, BB, and MS-H wrote sections of the manuscript and took part in designing the study. MS-H was responsible for this study.

## Conflict of Interest Statement

The authors declare that the research was conducted in the absence of any commercial or financial relationships that could be construed as a potential conflict of interest.
