# Supplementary material for: Cell-Free Protein Synthesis From Fast-Growing Vibrio natriegens
Source: Front Microbiol. 2018 Jun 1;9:1146. doi: 10.3389/fmicb.2018.01146 (PMC5992293; doi:10.3389/fmicb.2018.01146)
Supplement: Supplementary file 1 [file Presentation_1.pdf]

Cell-free protein synthesis from fast-growing *Vibrio natriegens*

Authors

Jurek Failmezger[#], Steffen Scholz[#], Bastian Blombach, Martin Siemann-Herzberg*

Institute of Biochemical Engineering
University of Stuttgart
Stuttgart, Germany

[#] equal contribution
*Corresponding author
siemann@ibvt.uni-stuttgart.de (MSH)

Supplementary Material

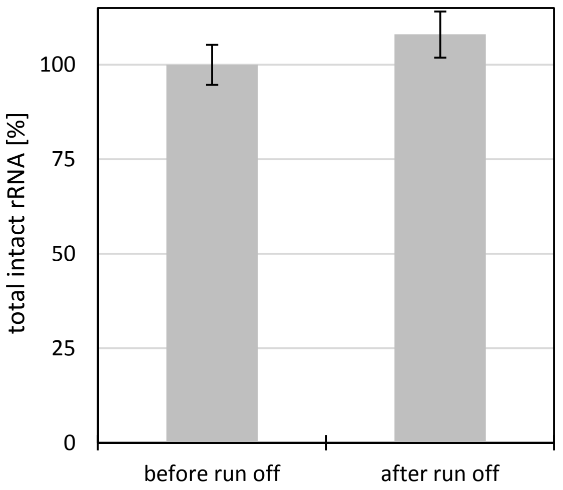

Figure S1: Amount of total intact rRNA determined before and after the run-off procedure.

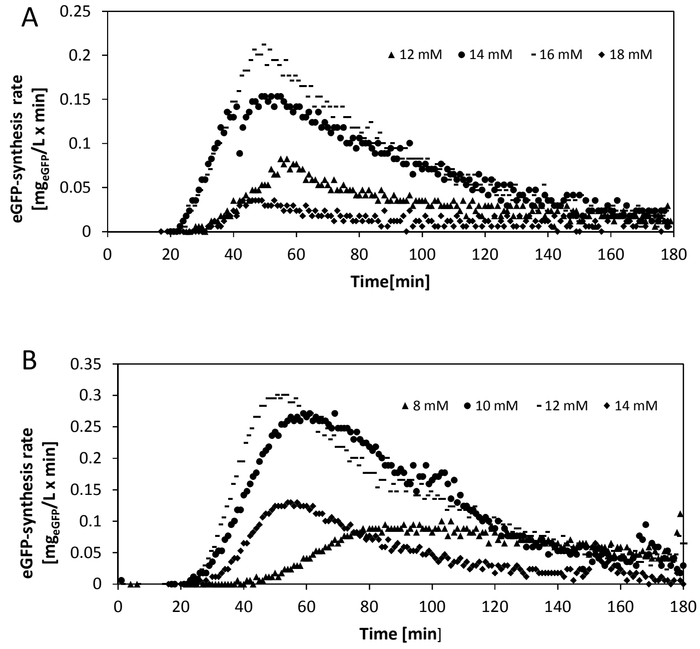

Figure S2: Cell-free protein synthesis by *Vibrio* fueled by **(A)** PEP and **(B)** pyruvate. Shown are the differential volumetric synthesis rates over the reaction time of eGFP at different concentrations of Mg^{2+}.

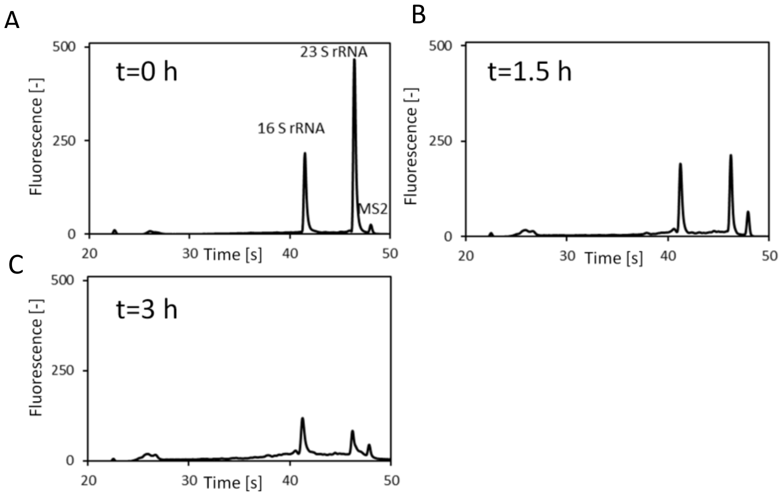

Figure S3: rRNA analysis by capillary gel electrophoresis with laser-induced fluorescence detection during a CFPS reaction fueled by creatine phosphate. Shown are the respective peaks for the 16S rRNA, 23S rRNA, and MS2 RNA (internal standard) at the start of the reaction **(A)**, after 1.5 h **(B)** and after 3 h **(C)**.
